# Remembering people with dementia during the COVID-19 crisis

**DOI:** 10.12688/hrbopenres.13030.2

**Published:** 2020-05-28

**Authors:** Eamon O'Shea

**Affiliations:** 1 National University of Ireland Galway, Galway, Ireland

**Keywords:** Dementia, COVID-19, crisis, response

## Abstract

This letter argues that we need to pay particular attention to people with dementia during this difficult time of the COVID-19 pandemic. Social distancing rules and cocooning for people aged 70 years and over are now in place in Ireland to slow down the rate of infection and protect vulnerable older people.  This letter argues that we need, more than ever, to assert the personhood of people with dementia at this difficult time. That means more person-centred care and practical support structures for family carers to allow them to continue to care at home in a safe and life-enhancing way. New public broadcasting initiatives could create information and communication channels for people with dementia and their carers, as well as demonstrating empathy and solidarity with their predicament. Government, the Department of Health, the HSE and the voluntary sector have risen to the challenge of COVID-19 in all sectors of society. So too have ordinary citizens. Now we need to unite even more to create an unyielding commitment and adherence to the principles of  decency, justice  and equity in the allocation of scarce  health and social care resources. By doing this, we will demonstrate our caring potential and capacity in a way that reflects our shared humanity, not only in the current crisis, but into the future.

## Disclaimer

The views expressed in this article are those of the author(s). Publication in HRB Open Research does not imply endorsement by the Health Research Board of Ireland.

Things have changed and likely changed forever. We are in the throes of the biggest international crisis since the Second World War. Domestically, our lives have been turned upside down in the space of a few short weeks. At the time of initial submission of this letter (6 ^th^ April, 2020), there were 5,364 cases of COVID-19 in Ireland and 174 people had died since the initial outbreak of the disease in the country ( Irish Government News Service, 2020). By final refereed submission (15 ^th^ May, 2020), these numbers had increased to 23,827 cases and 1,506 deaths respectively. An unprecedented lockdown is in place in the country, with citizens being confined to their homes in an effort to slow down the rate of infection. People aged 70 years and over are seen as particularly vulnerable to COVID-19 and therefore are the subject of specific national ‘cocooning’ measures designed to keep them safe but apart from their families and from the rest of the community. These measures are hard for all citizens and for older people in particular, but they are especially difficult for people with dementia, the majority of whom are likely to be 70 years and over. Dementia is a progressive and debilitating neurodegenerative condition which significantly impacts on memory, understanding and quality of life, as well as an individual’s ability to continue living independently.

There are currently an estimated 55,266 people with dementia in Ireland; 34,818 of whom are estimated to be living at home in the community ( O’Shea *et al.*, 2017). Many of these people may not even be aware that they have dementia and some of them will not be in regular contact with the health and social care system. Therefore, some people with dementia may be particularly vulnerable to COVID-19 infection, particularly if they have not heard about the disease, have heard but have forgotten, or have not understood the messages of cocooning and self-isolation coming from the government and the Health Service Executive (HSE). Therefore, we need to continuously reinforce the basic health messages of hand-washing, hygiene and social distancing for people with dementia and support them with these activities. That is the responsibility of everyone of us, not just family carers, the government and the HSE.

Just over 20,000 people with dementia live in residential accommodation, the vast majority of whom live in long-stay facilities, mainly in nursing homes ( O’Shea *et al.*, 2017). From the outset, these people faced a worrying and uncertain future, as evident by the significant number of clusters of the virus in care home settings. The initial focus of emergency planning on the acute care sector resulted in a belated prioritisation of residential care settings, which has been addressed over time. But there has been significant mortality in the residential care sector since this letter was first submitted, not only in Ireland but internationally ( Pierce *et al.*, 2020). This has focused renewed attention on optimal care settings for dependent older people, including the role, potential, funding and integration of the residential care sector in overall social care, something that needs to be addressed when the current crisis abates ( O’Shea *et al.*, 2019). In the meantime, person-centred care, based on the tenets of a personhood philosophy ( Kitwood & Bredin, 1992; Kitwood, 1997), remains the best approach to providing meaningful support for people with dementia in residential care facilities. Protecting and respecting people with dementia, knowing their likes and dislikes, offering sensory stimulation, providing meaningful psychosocial activities and keeping people connected (however virtual) is more important than ever ( Kane, 2001; Lawrence *et al.*, 2012).

There are an estimated 60,000 family carers in the community looking after someone with dementia in Ireland ( O’Shea *et al.*, 2017). Caring for a person with dementia compared with other caring roles places greater demands and strain on family members, at the best of times ( O’Shea, 2003). The family caregiver of a person with moderate to severe dementia in Ireland is likely to be providing round-the-clock care, sometimes struggling to cope with distressing challenging behaviours, leading to emotional and psychological strain even in loving relationships ( Livingston *et al.*, 2017). Carers are facing additional difficulties now, given the new rules on social distancing and cocooning. It is very difficult to provide care at a distance, especially for a person with dementia who may not fully, or consistently, understand the reason for that distancing. We all can support carers by staying in touch through our own networks and offering emotional sustenance, sometimes listening is enough, and by providing more practical support with shopping and medicine supplies. Empathy is an under-rated virtue in difficult times and can be easily displayed through a phone call or social media communication.

What type of things matter at this time for people with dementia? People with dementia and their carers, like all of us, have physical needs so exercise is important, both for people living at home and in residential care facilities ( World Health Organisation, 2020). People’s need for on-going emotional and psychological support should also not be under-estimated. We need to let people with dementia and family carers know how they can support and sustain themselves and that we are with them during the crisis. If we can have innovative new programmes for school-going children on national television, as is currently the case in Ireland ( https://www.rte.ie/learn/), why not provide similar weekly educational programmes for dependent older people and their family carers now living in the same social circumstances. It would be wonderful to see public broadcasting initiatives providing information and advice on physical exercise in the home, sensory stimulation activities, reminiscence-based ideas, brain health challenges, music therapies and creative opportunities for people with dementia ( Moniz-Cook & Manthorpe, 2009). Local radio could offer similar programmes, drawing on the expertise of HSE staff and the Alzheimer Society of Ireland (ASI). We need to be creative in the way we communicate with the dementia community and provide practical information and support to address physical and emotional needs.

There is a strong and vibrant voluntary and community ethos in Ireland. This can be very beneficial during the current difficulties, but it needs to be harnessed and co-ordinated, something that the government is now doing ( Irish Government News Service, 2020a). The ASI is doing powerful work in supporting people with dementia and their carers at this time and can be contacted at www.alzheimer.ie. They have a number of resources available to support people with dementia and family carers, particularly in relation to information, advice and helpful tips on how to deal with the COVID-19 crisis. Seniorline, ALONE and Age Action are other organisations that provide practical listening and support services for all older people that are invaluable at this time. In general, we need to make maximum use of both old and new technologies to connect with people with dementia and their carers and hopefully this current period will stimulate further innovation in regard to the use of technology ( Manthorpe & Moniz-Cook, 2020). Assistive devices can be used to maintain people’s independence and reduce worry for families, while digital reminiscence may be a means of promoting enjoyable activity in care settings ( Subramaniam & Woods, 2016; Moon & Park, 2020).

The government, HSE staff, private providers, nursing homes, voluntary organisations and families have been doing their very best in trying circumstances to look after people with dementia. What’s needed is for all of us to fully recognise the inherent personhood in people with dementia, especially their need for additional support, connectivity and communication during the crisis. This is the time when we must double up on our commitment to people with the disease, when we reassure them *that they will not be left behind,* to re-echo the words of our Minister for Health ( Department of Health, 2020). The message to citizens should be simple and straight-forward – social distancing and cocooning does not mean social isolation and the loss of citizenship for people with dementia. Human rights have never been more important, particularly the right to life, in all its forms, within the dementia population ( Cahill, 2019). Alzheimer Europe’s position paper on the allocation of scarce medical resources for intensive care services during the COVID-19 pandemic is, for example, firmly rooted in a rights-based approach ( Alzheimer Europe (3rd April 2020)). Similarly, their recommendations on promoting the well-being of people with dementia and carers during the pandemic reflects a holistic, person-centred approach to the provision of services and supports ( Alzheimer Europe (14th April 2020). This is the blueprint for a virtuous approach to addressing the needs of people with dementia and carers during, and after, the crisis.

In Ireland, we need a strong and unbreakable social bond that we will not ration available resources by age, condition or income, but will continue to act on the basis of carefully assessed individual need and circumstances. Inter-generational solidarity is the corner-stone of Irish social policy. It must be especially nourished and protected at this time of extreme crisis. Our collective voice now can be the voice of the person with dementia, an echo chamber for the voice of our parents and grand-parents, a promise to our future selves, one that is strong in terms of an adherence to the principles of decency, justice and equity. By doing this, we will be able to allocate resources and our caring potential in a way that reflects our shared humanity. There will be time enough in the future to learn the lessons of COVID-19 in relation to the delivery, organisation and financing of health and social care in the country. Now is not that time, as we muster to support our government and front-line staff in dealing with the crisis. In doing so, we must especially protect those who can no longer remember, otherwise we, ourselves, will never be forgotten or forgiven.

## Data availability

### Underlying data

No data are associated with this article.
