# Supplementary material for: Remembering people with dementia during the COVID-19 crisis
Source: HRB Open Res. 2020 May 28;3:15. Originally published 2020 Apr 16. [Version 2] doi: 10.12688/hrbopenres.13030.2 (PMC7195897; doi:10.12688/hrbopenres.13030.2)
Supplement: Peer review history

# Peer review: Reviewer response for version 2

**DOI:** 10.21956/hrbopenres.14163.r27466

**Published:** 2020-06-02

**Authors:** Brendan Walsh

**Affiliations:** 1 Economic and Social Research Institute (ESRI), Dublin, Ireland

Does the article adequately reference differing views and opinions?

Yes

Are all factual statements correct, and are statements and arguments made adequately supported by citations?

Yes

Is the Open Letter written in accessible language?

Yes

Where applicable, are recommendations and next steps explained clearly for others to follow?

Yes

Is the rationale for the Open Letter provided in sufficient detail?

Yes

Reviewer Expertise:

Health Economics. Inequalities. Health System Analysis. Cancer.

I confirm that I have read this submission and believe that I have an appropriate level of expertise to confirm that it is of an acceptable scientific standard.

# Peer review: Reviewer response for version 2

**DOI:** 10.21956/hrbopenres.14163.r27467

**Published:** 2020-05-29

**Authors:** Esme Moniz-Cook

**Affiliations:** 1 Psychology Ageing and Dementia Care Research, Faculty of Health Sciences, Department of Psychological Health, Wellbeing and Social Work, University of Hull, Hull, UK; 2 INTERDEM, Hull, UK

The author has done an excellent revision to include important perspectives relating to the human rights and technology in his revision as well as updating the paper to the present date. I can definitely approve this revised paper.

Does the article adequately reference differing views and opinions?

Partly

Are all factual statements correct, and are statements and arguments made adequately supported by citations?

Partly

Is the Open Letter written in accessible language?

Yes

Where applicable, are recommendations and next steps explained clearly for others to follow?

Yes

Is the rationale for the Open Letter provided in sufficient detail?

Yes

Reviewer Expertise:

NA

I confirm that I have read this submission and believe that I have an appropriate level of expertise to confirm that it is of an acceptable scientific standard.

# Peer review: Reviewer response for version 1

**DOI:** 10.21956/hrbopenres.14125.r27326

**Published:** 2020-05-11

**Authors:** Esme Moniz-Cook

**Affiliations:** 1 Psychology Ageing and Dementia Care Research, Faculty of Health Sciences, Department of Psychological Health, Wellbeing and Social Work, University of Hull, Hull, UK; 2 INTERDEM, Hull, UK

As cautious nuanced responses to emerging from the COVID-19 ‘ *lockdown’* begin, older people with dementia living in care homes may ‘ *have been left behind’* in some countries. Additionally, worldwide campaigns to raise dementia awareness (The Lancet, 2017 ^1^), has not overcome the pervasive influence of fear and nihilistic attitudes about dementia (Alzheimer’s disease International 2019 ^2^). This thoughtful letter, therefore, remains timely, beyond its published date of a month ago. It highlights how this pandemic could have an important role in stimulating societies to take practical steps to counteract the double stigma of age and dementia ( ^1^Moniz-Cook and Manthorpe 2009 p.16 ^3^) that continues to undermine person-centered care. To achieve this the author draws on the many creative evidence-based opportunities across Europe ( ^1^Moniz-Cook & Manthorpe 2009) to assist families to live as well as they can despite the disabilities of age and dementia. These have broadened in scope over the past decade, with flourishing technological approaches to dementia care (Manthorpe & Moniz-Cook 2020; Ch. 9 ^4^). As strategies to maintain physical distancing have become the first-line action in the battle against COVID-19, online, digital, and other forms of assistive technology the author could pay particular attention to these growing innovations. The ENABLE project in Ireland ( ^1^Moniz-Cook & Manthorpe 2009; Ch. 8) describes how assistive devices were used to maintain people’s independence and reduce worry for families and digital reminiscence is a means of promoting enjoyable activity in care settings (Subramaniam and Woods 2016 ^5^; Moon & Park 2020 ^6^). Today people with dementia living at home counteract the effect of physical distancing, with online social interaction ( http://www.innovationsindementia.org.uk/the-coronavirus-situation/).

 An important point about the role of the media (p3 para.5) and volunteers (p.3 para4.) is made. *The toddlers who took on dementia* ( https://www.bbc.co.uk/programmes/b0b3kk1h) demonstrates that the media can help to alter nihilistic public perception about psychosocial experiences of people with dementia. The call for harnessing co-ordination could, therefore, go further to counteract the pervasive stigma attached to dementia. For example, coordination of volunteer-led singing initiatives ( https://www.singireland.ie/news/sing-ireland-covid-19-update ) through television and other media; or clips of people enjoying outside ‘walking’ space with volunteers are emphases for recommendations made by this letter.

 Citizens living with dementia have learned much through their ongoing fight against the fear and uncertainty that travels with a dementia-diagnosis. Coordinated initiatives between volunteers and broadcasters could now offer them the opportunity to reciprocate, for example with visual media-led of how ‘moments’ (MacPherson *et al.* 2009 ^7^) of pleasure can encompass how people with dementia wish to live well (Øksnebjerg *et al.* 2018 ^8^) and contribute to their communities. The differing views about resources are missing. Perhaps the rights of people with dementia can be linked to Alzheimer Europe’s position statement to scarce medical care (3 ^rd^ April 2020) https://www.alzheimer-europe.org/Policy/Our-opinion-on/Triage-decisions-during-COVID-19-pandemic;/ recommendations for other care (14 April 2020) https://www.alzheimer-europe.org/Policy/Our-opinion-on/Wellbeing-of-people-with-dementia-during-COVID-19-pandemic.

^1^First author name quoted by the author of this letter (pg 3 para 5) is incorrect: it should read Moniz-Cook & Manthorpe in text/reference list: Moniz-Cook E.D & Manthorpe, J. Eds.

**Points to be addressed:**

1. Author name (p.3 para. 5) /reference list revision: Moniz-Cook
2. Emphasis: Technology for social interaction, perhaps connecting volunteering and media activity. 
3. Rights of people with dementia reflecting recommendations for better care; consider Alzheimer Europe’s statement on scarce medical care (3 ^rd^ April 2020) https://www.alzheimer-europe.org/Policy/Our-opinion-on/Triage-decisions-during-COVID-19-pandemic;/other care (14 April 2020) https://www.alzheimer-europe.org/Policy/Our-opinion-on/Wellbeing-of-people-with-dementia-during-COVID-19-pandemic.

Does the article adequately reference differing views and opinions?

Partly

Are all factual statements correct, and are statements and arguments made adequately supported by citations?

Partly

Is the Open Letter written in accessible language?

Yes

Where applicable, are recommendations and next steps explained clearly for others to follow?

Yes

Is the rationale for the Open Letter provided in sufficient detail?

Yes

Reviewer Expertise:

NA

I confirm that I have read this submission and believe that I have an appropriate level of expertise to confirm that it is of an acceptable scientific standard.

## References

[1] : Dementia burden coming into focus. *The Lancet*.2017;390(10113) : 10.1016/S0140-6736(17)33304-4 10.1016/S0140-6736(17)33304-4 29256397

[2] : World Alzheimer Report 2019: Attitudes to dementia. *Alzheimer’s Disease International*.2019; Reference source

[3] : Timely Psychosocial Interventions in Dementia Care; Evidence-Based Practice. *Jessica Kingsley Publishers*.2009;

[4] : Timely Psychosocial Interventions in Dementia Care; Evidence-Based Practice. *Jessica Kingsley Publishers*.2020;

[5] : Digital life storybooks for people with dementia living in care homes: an evaluation. *Clin Interv Aging*.2016;11: 10.2147/CIA.S111097 1263-1276 10.2147/CIA.S111097 27698556PMC5034922

[6] : The effect of digital reminiscence therapy on people with dementia: a pilot randomized controlled trial. *BMC Geriatr*.2020;20(1) : 10.1186/s12877-020-01563-2 166 10.1186/s12877-020-01563-2 32375661PMC7204054

[7] : An art gallery access programme for people with dementia: 'you do it for the moment'. *Aging Ment Health*.2009;13(5) : 10.1080/13607860902918207 744-52 10.1080/13607860902918207 19882413

[8] : Towards capturing meaningful outcomes for people with dementia in psychosocial intervention research: A pan-European consultation. *Health Expect*.21(6) : 10.1111/hex.12799 1056-1065 10.1111/hex.12799 29920881PMC6250864

# Response

**Published:** 2020-05-18

**Authors:** Eamon O'Shea

**Affiliations:** NUI Galway, Ireland

Thank you for this review. I have  briefly addressed the  issues you raised  in relation to references, rights and technology. Thank you also for the  additional references.

# Peer review: Reviewer response for version 1

**DOI:** 10.21956/hrbopenres.14125.r27327

**Published:** 2020-04-28

**Authors:** Brenda Gannon

**Affiliations:** 1 Centre for the Business and Economics of Health, University Of Queensland, Brisbane, Australia

This letter is timely and well warranted, given the escalation of the COVID-19 escalating. ‘No-one will be left behind’ must not be just a statement, but must be followed up with meaning. This is particularly important for more vulnerable people in society, such as those with dementia. The basic teaching of health economics, is to fairly distribute scarce resources, ensuring efficiency, equity and social justice are adhered to in decision making. This letter precisely makes this argument. In the case of people with dementia, if they don’t have their own voice, then their carers and society need to act, to ensure they are not left behind.

 The letter is written with sound theoretical foundations, ensuring the person is at the centre of care provided. The arguments are well set out and include appropriate references.

 The language is appropriate, considerate and respectful. People with dementia are people, not patients, not old people, not aged and so on. This is a very nice aspect of this letter, making it a pleasure to read. 

 The practical proposals for broadcasting are great. The implementation of such would need further work and assurance that any intergenerational viewing would not be derogatory or make a person feel less a person, and more so a statistic. This comment arises from coverage I have seen in Australia, of a new program on TV that shows older people and children undertaking basic exercises - encouraging people at home to follow these, so all age groups (but especially young children and older people) can connect and then exercise together virtually.

 While the idea is meant in good faith, the implementation requires way more thought on the theory behind it and how to demonstrate inclusivity, ability and not just categorise those aged 70 and over into one group, depicted as those who may be less mobile and frail. Many people aged 70 and over, and even 80 and over, are more agile than depicted in that programme. I hope RTE will bear this in mind and consult with relevant academics on implementing a fair and respectful broadcast.

 Of course since this letter was written, there has been a rapidly evolving situation and unfortunately the mortality has increased significantly with 55% being those in nursing homes. It would be great to see a continuation of this letter, and update, to show how many of those had dementia and how many were either in the community or the nursing homes.

 Lastly, social distancing is more vital than ever, but this can be done in a humane way, including people with dementia and also any other older people. If an older person is capable of going for a walk or their normal physical activity, then they should be allowed do so, as long as they respect the physical distance rules that apply to everyone, and ensure if they have any underlying condition that puts them at increased risk, then they should take that risk into consideration when planning their exercise. Overall, exercise and vitamin D are vital to good health and cocooning everyone, regardless of their level of frailty may therefore need to re-considered and the restriction loosened so that people can redeem their dignity and inclusion into society.

Does the article adequately reference differing views and opinions?

Yes

Are all factual statements correct, and are statements and arguments made adequately supported by citations?

Yes

Is the Open Letter written in accessible language?

Yes

Where applicable, are recommendations and next steps explained clearly for others to follow?

Yes

Is the rationale for the Open Letter provided in sufficient detail?

Yes

Reviewer Expertise:

NA

I confirm that I have read this submission and believe that I have an appropriate level of expertise to confirm that it is of an acceptable scientific standard.

# Response

**Published:** 2020-05-18

**Authors:** Eamon O'Shea

**Affiliations:** NUI Galway, Ireland

Thank you for this review. You have raised many important points. I have attempted to incorporate many of them in the final version, especially in regard to nursing homes.

# Peer review: Reviewer response for version 1

**DOI:** 10.21956/hrbopenres.14125.r27329

**Published:** 2020-04-27

**Authors:** Manuel Ruiz Adame Reina

**Affiliations:** 1 Department of Applied Economics, School of Social Sciences and Law, University of Granada, Melilla, Spain

This paper underlines a relevant issue in this moment. The need of a personhood approach in the case of people with dementia when protective measures are taken such as the isolation to fight against Covid-19. From this initial issue other important questions arise, the role of the different social stakeholders to provide support to the people with dementia and their caregiver and how to assure that this support could be maintained in the future, not only in the current crisis.

 This situation is completely new for everybody all over the world. Things have changed in a very short period of time and no health neither social system was ready to assume the stress test that a pandemic like this was going to be. In some countries such as Italy or Spain, for their demographic characteristics and for certain unsuccessful epidemiological measures, the number of people affected are proportionally greater than in Ireland, but in general, the Covid-19 has attacked with more aggressiveness to the older people, and mainly to people who live in nursing homes. 

 Isolation seems to be the most effective measure, but with people with dementia, as the author states, we must bear in mind the cases in which caregivers have to cope with behavioural disturbances, very common among fronto-temporal dementias and dementias with Lewy bodies. In those cases, the lockdown can be a very stressful situation for the people with dementia and for their caregivers.

 The author also states the case of people in very mild stages of the syndrome in which people still have a certain level of autonomy and can live on their own. These people are a special group in a moment in which the authorities command social distant measures that maybe they do not understand.

 On the other hand, the author points out the role of the caregivers. They have to adapt to new circumstances, and maybe they do not have enough information about how to do it. The local authorities and the media can be a great support for them. Educational programs such as those proposed by the author could be very helpful.

 From my point of view, this paper is a very interesting work that resumes some of the most relevant worries among caregivers, health and social stakeholders. It could very useful for policy makers. It merits to be indexed.

**Points to be addressed:**

 The number of cases could be actualised in the final version at the time of publication.

 Even though it is very widely used the term “social distancing”, in my opinion is more accurate to use “physical distancing”. The first one leads to thinking in emotional distancing, or even in a reduction of empathy with people with dementia and their caregivers.

   

 It also could be interested to include (if possible) questions such as :

- How this situation affects to the caregivers in their working situation?
- How other family members change their attitude to give support to the main caregiver under these circumstances?
- How different clinical profiles (E.g. behavioural problems) can modify the need of support? 
- Did the caregivers have enough access to protection equipment (E.g. gloves, masks…)?

Does the article adequately reference differing views and opinions?

Yes

Are all factual statements correct, and are statements and arguments made adequately supported by citations?

Yes

Is the Open Letter written in accessible language?

Yes

Where applicable, are recommendations and next steps explained clearly for others to follow?

Yes

Is the rationale for the Open Letter provided in sufficient detail?

Yes

Reviewer Expertise:

Health Economics. Dementias. Long Term Care. Social costs.

I confirm that I have read this submission and believe that I have an appropriate level of expertise to confirm that it is of an acceptable scientific standard.

# Response

**Published:** 2020-05-18

**Authors:** Eamon O'Shea

**Affiliations:** NUI Galway, Ireland

Thank you for your comments. I have briefly  highlighted some of the carer issues highlighted by you in your review. They are all important questions that deserve a paper of their own -  see paper by Phillips et al  in HRB Open.

# Peer review: Reviewer response for version 1

**DOI:** 10.21956/hrbopenres.14125.r27328

**Published:** 2020-04-20

**Authors:** Brendan Walsh

**Affiliations:** 1 Economic and Social Research Institute (ESRI), Dublin, Ireland

Covid-19 affects the older and more vulnerable populations the most. This letter focuses on people living with dementia. This is a group with a higher susceptibility to the illness itself. This group has also seen major changes to their lives deemed necessary to try to negate the probability of infection with the virus, such as cocooning. It is increasingly clear within Ireland and other countries that long-term residential care homes have become epicentres of the infection. As many of these homes in Ireland cater almost exclusively for dementia patients, or are the residence for many people living with dementia, the ramifications of the Covid for people living with dementia are far reaching.

 This is a timely article highlighting how the health system, and broader society, may help this population during this crisis. There will be many research pieces on the provision of medical care to certain population groups forthcoming in the research literature. The straightforward recommendations in this piece also provide some simple changes that could be easily implemented. But these changes or policies are to stimulate the ‘person’, rather than treat the ‘patient’, and provide some normalcy to their everyday lives. These points, and the discussion of wider society values on living with vulnerable populations are to be welcomed.

**Points to be addressed:**

 The numbers of cases are changing daily. But as the prevalence of Covid has increased substantially since the time of writing (April 6th), especially in the residential care sector (with over 40% of nursing homes for examples having a Covid case), a greater recognition of this issue and a more updated number on Covid in the opening paragraph is recommended.

Does the article adequately reference differing views and opinions?

Yes

Are all factual statements correct, and are statements and arguments made adequately supported by citations?

Yes

Is the Open Letter written in accessible language?

Yes

Where applicable, are recommendations and next steps explained clearly for others to follow?

Yes

Is the rationale for the Open Letter provided in sufficient detail?

Yes

Reviewer Expertise:

Health Economics. Inequalities. Health System Analysis. Cancer.

I confirm that I have read this submission and believe that I have an appropriate level of expertise to confirm that it is of an acceptable scientific standard.

# Response

**Published:** 2020-05-18

**Authors:** Eamon O'Shea

**Affiliations:** NUI Galway, Ireland

Thank you for  your comments. I have updated case numbers and mortality data.
